# Genetics of Late-Onset Alzheimer's Disease: Update from the Alzgene Database and Analysis of Shared Pathways

**DOI:** 10.4061/2011/832379

**Published:** 2011-12-10

**Authors:** Paolo Olgiati, Antonis M. Politis, George N. Papadimitriou, Diana De Ronchi, Alessandro Serretti

**Affiliations:** ^1^Institute of Psychiatry, University of Bologna, Viale Carlo Pepoli 5, 40123 Bologna, Italy; ^2^1st Department of Psychiatry, National and Kapodistrian University of Athens Medical School, Eginition Hospital, 10679 Athens, Greece

## Abstract

The genetics of late-onset Alzheimer's disease (LOAD) has taken impressive steps forwards in the last few years. To date, more than six-hundred genes have been linked to the disorder. However, only a minority of them are supported by a sufficient level of evidence. This review focused on such genes and analyzed shared biological pathways. Genetic markers were selected from a web-based collection (Alzgene). For each SNP in the database, it was possible to perform a meta-analysis. The quality of studies was assessed using criteria such as size of research samples, heterogeneity across studies, and protection from publication bias. This produced a list of 15 top-rated genes: *APOE, CLU, PICALM, EXOC3L2, BIN1, CR1, SORL1, TNK1, IL8, LDLR, CST3, CHRNB2, SORCS1, TNF,* and *CCR2.* A systematic analysis of gene ontology terms associated with each marker showed that most genes were implicated in cholesterol metabolism, intracellular transport of beta-amyloid precursor, and autophagy of damaged organelles. Moreover, the impact of these genes on complement cascade and cytokine production highlights the role of inflammatory response in AD pathogenesis. Gene-gene and gene-environment interactions are prominent issues in AD genetics, but they are not specifically featured in the Alzgene database.

## 1. Introduction

Alzheimer's disease (AD) is the leading cause of dementia in developed countries. It afflicts 5.3 million individuals in the US. Total direct and indirect cost is US$ 172 billion per year [1]. The prevalence of AD shows an age-dependent progression in the elderly. Thus, approximately 5% of all persons over age 70 have AD (late-onset AD, LOAD); this proportion raises to 25%–45% in “oldest old” (>85 years) individuals. About 10% of AD patients develop symptoms before age 65, more often in their 40 s or 50 s [1]. 

Clinically, AD is characterized by progressive impairments in memory and other cognitive domains. Behavioral and psychiatric symptoms (BPSDs), clustered into agitation/aggression, mood disorders, and psychosis, may occur with disease progression [2]. Neuroimaging studies display atrophy in the cerebral cortex and the hippocampus of AD brain [3, 4]. A marked neural loss is reported in cholinergic nuclei in the basal forebrain as opposed to an overactivation of NMDA-mediated glutamatergic pathways [5]. Postmortem examination reveals the neuropathological hallmarks of AD that include neuritic plaques, neurofibrillary tangles (NFTs), and amyloid angiopathy [6]. Neuritic plaques are extracellular aggregates of beta(**β**)-amyloid protein in a milieu of reactive astrocytes and activated microglia. NFTs are intraneuronal cytoplasmatic filaments composed of hyperphosphorylated tau, frequently conjugated with ubiquitin. Pathophysiologically, researchers assign a pivotal role to beta-amyloid deposition in the brain [6]. Beta-amyloid peptides are derived from proteolytic activity of proteinases (*β* and *γ* secretases) on amyloid precursor protein (APP). Study performed in transgenic animals suggest that neuroinflammation plays an important role in the process of cerebral amyloid deposition [7]. It has been shown that inflammatory cytokines such as interleukin (IL)-1*β*, IL-6, tumor necrosis factor-*α*gTNF-*α*), or IFN gamma can augment APP expression and A*β* formation [8, 9]. It was also reported that nonsteroidal anti-inflammatory drugs are able to transcriptionally upregulate *β*-secretase mRNA, protein and enzymatic activity [10]. Intraperitoneal injection of lipopolysaccharide induced memory impairment in mice associated with amyloidogenesis [11]. On the other hand, recent lines of evidence indicate that blood-borne mononuclear phagocytes are capable of infiltrating the brain and restricting beta-amyloid plaques, thereby, limiting disease progression. Indeed, there would be two types of monocytes. M1 has proinflammatory effects detrimental to AD brain. Alternately, M2 macrophages are recruited to noninflamed tissues and are believed to be highly phagocytic, anti-inflammatory effector cells. They could clear beta amyloid via phagocytosis [12]. APP follows a complex intracellular trafficking pathway that influences its processing to either a soluble fragment (sAPP*α*) or to sAPP*β* and the insoluble A*β* [13]. The cleavage of APP to generate pathological A*β* may occur when APP transits from the endosome to the lysosome. This is associated with APP gene mutations, whereas wild APP has rapid and direct transport from the cell surface to the lysosomes [14]. APP trafficking is regulated by sorting-protein-related receptor (sorLA), which binds the APP in the Golgi reducing the availability of precursors for transport, cleavage, and transformation into A*β* [15, 16]. Over the last few years, a shift occurred in research focus from amyloid deposition to tauopathy. The physiological function for protein tau is binding to and stabilization of microtubules. Microtubules ensure cell shape and constitute roads of transport. Microtubule-dependent transport is ensured by families of motor proteins dyneins and kinesins, respectively, for retrograde transport from distal processes towards soma and as plus-end directed motor for anterograde transport. The effect of protein tau on transport appears to be dual. First, hyperphosphorylation can cause protein tau to detach from the microtubules and decrease its ability to control microtubule dynamics. On the other hand, increased levels of protein tau can saturate microtubules and hinder the “foot-stepping” of the motor proteins needed for axonal and dendritic transport. Both aspects of tau-related transport deficits have been observed and both can fit into a model leading to “starving synapses” that eventually culminates in neuronal death [17, 18]. It is acknowledged that 75% of people with AD have sporadic AD. This is most likely a multifactorial condition, which involves a combination of genetic, lifestyle, and environmental factors. 25% is familial AD (FAD). Early-onset AD encompasses 5% of FAD cases. Early-onset FAD is inherited in an autosomal dominant manner and is caused by mutations in one of these three genes: APP, PSEN1, and PSEN2. As for LOAD, the only established genetic factor is apolipoprotein E (APOE). The APOE gene is at chromosome location 19q13.2. APOE-associated Alzheimer's disease is due to a specific variation in the APOE gene called e4 allele. It is estimated that 40–65% of AD patients have at least one copy of the e4 allele [19]. Individuals with two e4 alleles have up to 20 times the risk of developing AD [19]. Another variant of the APOE gene, e2, has protective effects against the development of AD [20, 21]. Nonetheless, a third of patients with AD are *ApoE4* negative, and some *ApoE4* homozygotes never develop the disease. Since the early 90 s, more than six hundred genes have been investigated as susceptibility factors for LOAD (http://www.Alzgene.org/). We reviewed the best established LOAD genes and suggested a method to identify shared biological pathways. 

## 2. Methods

We used the AlzGene database to identify those genes that had the strongest association with LOAD, for which there was a qualitatively high level of evidence. AlzGene is a web-based synopsis of published association studies on AD [22]. AlzGene is regularly updated by studies retrieved from peer-reviewed journals and available in English language. Authors are encouraged to submit their data as soon as their work is accepted for publication. Data presented only in abstract form are not included. For all polymorphisms with minor allele frequencies in healthy controls >1%, and for which case-control genotype data are available in at least four independent samples, a meta-analysis is performed. Summary OR and 95 percent CIs are calculated using the DerSimonian and Laird random-effects model [23]. Genes which contain variants showing at least one significant OR in meta-analysis are included in a “Top Results” list. To establish their ranking, each positive meta-analysis is graded according to human genome epidemiology network (HuGENet) interim criteria for the assessment of cumulative evidence of genetic associations [24]. These criteria take into account the amount of evidence (sample size, measured as total number of minor alleles of cases and controls combined in the meta-analysis “*N* minor”; grade A: *N* minor exceeds 1,000; grade B: *N* minor is between 100 and 1,000; grade C: *N* minor is <100), consistency of replication (heterogeneity across studies, measured as I2; grade A: I2 point estimates <25%; grade B: between 25% and 50%; grade C: >50%) and protection from bias (the following potential reasons for bias in the meta-analysis results are assessed: summary OR < 1.15 (low OR); loss of significance after exclusion of first study; loss of significance after exclusion of studies with deviations from HWE in control groups; evidence for publication bias; grade A: no bias; grade B: no demonstrable bias, but important information is missing for its appraisal; grade C: evidence for clear bias that can invalidate the association). Overall epidemiologic credibility is graded as “A” (= strong) if associations received three A grades, “B” (= moderate) if they received at least on B grade but no C grades, and “C” (= weak) if they received a C grade in any of the three assessment fields. Loci with the same grade are ordered by *P*-value.

Genes with strong (A) and moderate (B) associations were included in a query set of the gene ontology database AmiGO to discover shared biological functions. The Gene Ontology [GO (http://www.geneontology.org/); Gene Ontology Consortium, 2000] project develops structured controlled vocabularies, or ontologies, to describe fundamental characteristics of genes and their products in a species-independent manner. Members of the GO consortium submit annotations made using these ontologies to the GO database for integration and dissemination. AmiGO (version 1.7) (http://amigo.geneontology.org/) is a web-based application that allows users to search, sort, analyze, visualize, and download data about gene ontologies and products [25]. Ontologies are clustered into three categories: (1) biological process: any process specifically pertinent to the functioning of integrated living units: cells, tissues, organs, and organisms. A process is a collection of molecular events with a defined beginning and end; (2) cellular component: the part of a cell or its extracellular environment in which a gene product is located. A gene product may be located in one or more parts of a cell, and its location may be as specific as a particular macromolecular complex, that is, a stable, persistent association of macromolecules that function together. (3) Molecular function: elemental activities describing the actions of a gene product at the molecular level. A given gene product may exhibit one or more molecular functions. The GO “Term Enrichment” tool, which determines whether the observed level of annotation for a group of genes (test dataset) is significant in the context of a background set, was useful for discovering relationships between genes. We included LOAD genes in test dataset; the UniProt Knowledgebase (UniProtKB: http://www.uniprot.org/), a large collection of gene products, was used as comparison dataset. Significant annotations were associated with at least two genes and more represented in test dataset (*P* = 0.003 after Bonferroni correction). Subsequently, the GO slimmer tool enabled to remap granular, specific annotations up to a user-specified set of high-level terms. We excluded terms that were nonspecific (e.g., “biological regulation”) or shared by less than three genes. Synonymous annotations (e.g., “cell death” and “regulation of cell death”) were collapsed into one term.

## 3. Results

The Alzgene database (updated 13 September 2010) includes 1,380 studies and 666 genes. The number of meta-analyses is 380. 

### 3.1. Top Genes Associated with LOAD

Forty-two genes have at least one positive meta-analysis (see Table 1). Of them, fifteen are supported by an adequate level of evidence (“A” or “B” grade on overall association credibility). These genes are reported below (meta-analysis results are referred to the best SNP for each gene).

**APOE**

The gene encoding apolipoprotein E (chromosome 19q 13.2) was associated with AD in thirty-eight case-control samples (Caucasian = 28; Asian = 4; African descent = 2; Hispanic descent = 1; mixed ethnic groups = 3) and four family-based studies. Overall OR was 3.77 (95% CI 3.29–4.32; I^2^ = 13) in Caucasian samples and 3.99 (95% CI: 2.86–5.57 I^2^ = 20) in Asian samples.

**CLU**

Clusterin (apolipoprotein J) is a chaperone molecule that appears to be involved in membrane recycling and apoptosis. Clusterin, like apolipoprotein E, is found in amyloid plaques [26]. Clusterin interacts with the soluble form of beta amyloid in animal models of AD and binds soluble beta-amyloid in a specific and reversible manner, forming complexes that cross the blood-brain barrier [27]. The gene-encoding clusterin* CLU* (chromosome 8) was investigated as a susceptibility factor for LOAD in genome-wide association studies (GWASs) [28–32] as well as candidate gene studies [33, 34]. Meta-analytic data reveal four polymorphisms consistently associated with LOAD. The best SNP is rs11136000 (Caucasian subjects; *N* minor = 53,712; OR 0.88 95% CI: 0.86–0.91). Valproic acid has been recently demonstrated to stimulate clusterin expression [35]. Plasma levels of clusterin have been recently associated with atrophy of the entorhinal cortex, baseline disease severity, and rapid clinical progression in AD [36]. These findings should prompt further investigations to ascertain the impact of CLU on AD phenotypes.

**PICALM**

Phosphatidylinositol-binding clathrin assembly proteins is a key component of clathrin-mediated endocytosis. It recruits clathrin and adaptor protein 2 (AP-2) to the plasma membrane and, along with AP-2, recognizes target protein. The attached clathrin triskelions cause membrane deformation around the target proteins enclosing them within clathrin-coated vesicles [37]. Of relevance to AD, PICALM appears to be involved in VAMP2 trafficking, a process that is crucial to the functional integrity of synapses. [38]. AD brains show a reduced number of synapses, and this reduction could correlate to cognitive defects better than the accumulation of plaques and tangles [39]. Alternatively, APP is processed in endocytotic compartments [40]; thus PICALM could promote the synthesis of beta amyloid by regulating endocytosis. The *PICALM* gene (chromosome 11) was analyzed in seven case-control samples [29–31, 33, 41, 42]. Overall *PICALM* was investigated in 10,251 patients and 18,270 controls. Two SNPs were associated with LOAD. The best one is rs3851179 (Caucasian subjects; *N* minor = 44,358 OR 0.88 95% CI: 0.85–0.91).

**EXOC3L2**

Exocyst complex component 3-like 2 is also involved in vesicle targeting during exocytosis of proteins and lipids that is essential to neuron outgrowth and integrity [43]. Seshadri et al. [30] reported an association between the *EXOC3L2* gene and LOAD in their multisample GWAS which included 1,140 patients and 1,210 controls (rs597668 *N* minor = 13,519 OR 1.17 95% CI: 1.12–1.23).

**BIN1**

Bridging integrator 1 is a member of the BAR adapter family which has been implicated in endocytosis and intracellular endosome trafficking [44, 45]. In addition, bin1 is crucial for the function of pathways leading to cell senescence and apoptosis [46–48]. The *BIN1* gene (chromosome 2) was investigated in four case-control samples [29, 30, 41] consisting of 4,473 patients and 7,659 controls. A meta-analysis showed that *BIN1* rs 744373 SNP was associated with LOAD (*N* minor = 24,713 OR 1.15 95% CI: 1.10 1.20).

**CR1**

Complement component receptor 1 regulates complement cascade via the inhibition of both classical and alternative pathway C3 and C5 convertases [49]. Notably, complement inhibition was shown to reduce the clearance of beta amyloid in animal models [50]. More recently, cr1 has been found to bind peripheral blood beta amyloid in a complement C3b-dependent manner, a mechanism that is implicated in the clearance of pathogens and proteins from the bloodstream. Levels of beta amyloid targeted by this pathway differed significantly in AD compared to mild cognitive impairment and nondemented elderly controls [51]. The gene encoding cr1, chromosome 1 (*CR1*), was investigated in eleven independent samples [28, 29, 31, 33, 41, 42, 52]. Overall sample included 13,193 cases and 20,551 controls. Meta-analytic data showed the association between* CR1* rs3818361 and LOAD in Caucasian subjects (*N* minor = 18, 779 OR 1.14 95% CI: 1.08 1.20).

**SORL1**

Sortilin-related receptor (SorLA) is a sorting receptor that regulates trafficking and processing of APP. SorLA acts as a retention factor for APP in trans-Golgi compartments/trans-Golgi network, preventing the release of the precursor into regular processing pathways [16, 53]. In addition, SorLA is an apolipoprotein E receptor (LR11). The gene-encoding SorLA, *SORL1*, was investigated as a susceptibility factor for LOAD in twenty-one case-control samples [54–74]. Nine SNPs were significantly associated with LOAD. The best one is rs2282649 (*N* minor = 1,734 OR 1.10 95% CI: 1.02–1.17). A recent study has suggested that the role of *SORL1* as a LOAD gene might be gender dependent, consistently demonstrated in women [67]. We confirmed this result in a sample of AD patients attending our center in Athens. In addition, we reported correlations between *SORL1* SNPs, psychosis, and proinflammatory cytokines [75].

**TNK1**

Nonreceptor tyrosine kinase 1 is involved in intracellular transduction pathways, and it was shown to enable TNF-alpha-induced apoptosis [76]. One polymorphism (rs1554948) in the *TNK1* gene (chromosome 17) was investigated in five samples [77], and it proved its association with LOAD (rs1554498; *N* minor = 3,538; OR 0.84 95% CI: 0.76–0.93).

**IL8**

Interleukin 8 is a proinflammatory cytokines. Cerebrospinal fluid levels of IL-8 were found to be increased in AD and mild cognitive impairment [78]. IL-8 production can be enhanced by beta amyloid [79]. The gene-encoding IL-8 (chromosome 4) was analyzed in four case-control samples [32, 80–82], including 660 patients and 933 controls, and it proved to be significantly associated with LOAD (rs4073; *N* minor = 1,157; OR 1.27 25%CI: 1.08–1.50). *IL8* showed gene-gene interactions with the methylenetetrahydrofolate reductase (MTHFR) [82] and interleukin-1alpha (ILalpha) [80] genes. Metal ions (zinc; copper) appear to play an important role in AD pathophysiology. For instance, the provision of a zinc-enriched diet was found to enhance Alzheimer-like spatial memory impairments in transgenic mice and to modify hippocampal deposits of amyloid plaques [83]. Zinc ions promote beta-amyloid aggregation leading to conformational changes [84]. A consistent amount of evidence links zinc and *IL8* pathways. Zinc deficiency increases the expression of cytokine-related genes (*TNF; IL1B; IL8*) in leukemia cell-lines. Elevated levels of pro-inflammatory cytokines including IL-8 were reported in a group of healthy old subjects coupled with low circulating levels of zinc [85]. Traumatic brain injury, a known risk factor for AD development [86], can modify the expression of proinflammatory cytokines [87].

**LDLR**

Low density lipoprotein receptor is implicated in cholesterol metabolism via endocytosis. Recently, it has been discovered that overexpression of brain LDLR is associated with decrease in APOE levels and beta amyloid due to either inhibited deposition or enhanced clearance [88]. Moreover, two members of the LDLR family were found to modulate APP trafficking [89]. The *LDLR* gene is localized to chromosome 19. Its association with LOAD was explored in twelve studies [32, 52, 90–99]. A polymorphism (rs 5930) showed a consistent association with LOAD (*N* minor = 1,228; OR 0.85 95% CI: 0.72–0.99). Zou et al. reported a sex modulation of *LDLR* gene that was linked to LOAD in male subgroup [99].

**CST3**

Cystatin C, a potent inhibitor of lysosomal proteinases, was shown to bind beta amyloid and to prevent beta-amyloid aggregation and deposition in mouse models [100]. More recently, cystatin levels have been positively correlated with beta-amyloid and tau protein in cerebrospinal fluid of individuals with AD, mild cognitive impairment, and healthy controls [101]. The gene-encoding cystatin, chromosome 20 (*CST3*), was associated with dementia in Lewy body disease. The association between *CST3* and LOAD was analyzed in fourteen Caucasian [32, 52, 72, 96, 102–111] and four Asian studies [112–115]. Overall Caucasian sample included 2,502 patients and 1,897 controls. Overall Asian sample included 814 patients and 1,293 controls. Two *CST3* polymorphisms were associated with LOAD in Caucasian groups. The best one is rs1064039 (*N* minor = 1,203; OR 1.16 95% CI: 1.00–1.33).

**CHRNB2**

Each nAChR protein is made up of a combination of five subunits, usually two alpha (*α*) and three beta (*β*) subunits. Many different combinations are possible, and the characteristics of each nAChR protein depend on which subunits it contains. In the brain, nAChR proteins most commonly consist of two *α*4 subunits and three *β*2 subunits. The* CHRNB2 *gene (chromosome 1) is responsible for producing the *β*2 subunit. A wide range of brain functions depend on nAChR channels, including sleep and arousal, fatigue, anxiety, attention, pain perception, and memory. The channels are also active before birth, which suggests that they are involved in early brain development. The association between *CHRNB2 *and LOAD was originally investigated in an Asian sample of 58 patients and 51 controls with negative results [116]. Three Caucasian studies followed the first one [117–119]. Cook et al. analyzed three samples and reported a significant association with LOAD (*N* minor = 227; OR 0.69 95% CI: 0.51–0.95).

**SORCS1**

SorCS proteins (like SorLA) are members of the Vps10p family of sorting receptors. SorCS1 binds to nerve growth factor (NGF) propeptide. Pro-NGF is increased in AD brains, and its binding to neurotrophin receptor p75 induces apoptotic cell death in neurons [120]. In addition, SorCS1 was involved in APP processing [121]. The gene-encoding SorCS1, *SORCS1* (chromosome 10), has been associated with insulin signaling and diabetes mellitus [122]. Grupe et al. [77] reported an association between *SORCS1 *and LOAD in four Caucasian samples (rs600879; *N* minor = 567; OR 1.34 95% CI: 1.09–1.65). A family-based study showed an association between *SORCS1 *and LOAD in a women subgroup [123].

**TNF**

Tumor necrosis factor alpha induces the production of beta amyloid [9], and it increases the risk of developing AD in cognitively intact elderly subjects [124]. The gene-encoding TNF (chromosome 6) has been extensively investigated as a susceptibility AD gene. One SNP (rs4647198) was significantly associated with LOAD in Asian populations [125–127] (*N* minor = 301; OR 1.37 95% CI: 1.05–1.08). Increase in the serum levels of TNF-alpha following acute inflammatory events was found to correlate with a 2-fold increase in the rate of cognitive decline over a 6-month period in AD patients. In addition, the rate of cognitive decline was four fold increased in patients with high basal levels of TNF-alpha [128]. Etanercept, a biological antagonist of TNF-alpha, is under evaluation as a therapeutic agent for AD. A rapid improvement in cognitive performance was reported following etanercept administration in a pilot study [129].

**CCR2**

Chemokine receptor 2 is IL-8 receptor. It is coupled with MAP-kinase pathway to modulate signaling transduction. *CCR2* gene (chromosome 3) was associated with LOAD in Caucasian samples [32, 130, 131] (rs1799864; *N* minor = 308; OR 0.73 95% CI: 0.56–0.97).

### 3.2. Shared Biological Pathways

Gene ontology analysis identified 146 GO terms more represented in test dataset (LOAD genes) than in UniProtKb collection (*P* < 0.001). Most of them were excluded or collapsed based on criteria reported above. The following terms were selected: “immune system process” (*TNF, IL8, CR1, CLU, CCR2, PICALM, *and* CHRNB2*); “vesicle-mediated transport” (*PICALM, SORL1, APOE, BIN1, LDLR, *and* CLU*); “cellular membrane organization” (*SORL1, APOE, PICALM, BIN1, *and *LDLR*); “alcohol metabolic process” (*CHRNB2, SORL1, APOE, TNF, *and* LDLR)*; “lipid transport” (*SORL1, APOE, LDLR, CLU, *and* TNF*); “steroid metabolic process” (*SORL1, APOE, TNF,* and* LDLR*); “cholesterol metabolic process” (*APOE, CLU, LDLR, *and* SORL1*); “cell death/apoptosis” (*APOE, TNF, *and *CLU*); “cell migration” (*IL8, APOE, *and* TNF*).

## 4. Discussion

The genetics of late-onset AD is a complex one. More than six-hundred genes have been investigated as susceptibility factors. They represent 2.9% of all genes with known function (http://www.geneontology.org/). This review focused on fifteen genes that have been consistently associated with LOAD in recent years. Such genes, however, participate in multiple functions, and it is difficult to discriminate which are pathophysiologically meaningful. Gene ontology (GO) is a synoptic way to annotate all functions amenable to a single gene or gene product. We performed a GO analysis of aforementioned genes to identify biological pathways common to all or most of them. In doing so, we discovered that those genes converged onto few pathways that are discussed below.

### 4.1. Cholesterol Metabolism

Five genes of our compilation (*APOE; LDLR; SORL1; CLU; TNF*) were implicated in lipid metabolism (four in cholesterol metabolism). This is consistent with epidemiological findings that show how having high cholesterol levels in midlife is a risk factor for developing AD in late life [132]. Beta amyloid is an intrinsically disordered protein (IDP) that lacks a well-defined 3D structure, but it undergoes a series of lipid-dependent conformational changes in membrane bilayers. Membrane-bound monomers are transformed into oligomers of varying toxicity rich in beta-sheet structures (annular pores; amyloid fibrils) or in alpha-helix structures (transmembrane channels) [133]. Condensed membrane nano- or microdomains (*lipid rafts)* formed by sphingolipids and cholesterol are privileged sites for the binding and oligomerisation of amyloidogenic proteins. By controlling the balance between unstructured monomers and *α* or *β* conformers (the chaperone effect), sphingolipids can either inhibit or stimulate the oligomerisation of amyloidogenic proteins [134]. Cholesterol has a dual role: regulation of protein-sphingolipid interactions through a fine tuning of sphingolipid conformation (indirect effect) and facilitation of pore (or channel) formation through direct binding to amyloidogenic proteins [134]. In view of a key role of cholesterol in beta-amyloid neurotoxicity, statins are currently under evaluation as potentially effective treatment for AD. Recently, a meta-analysis of three randomized trials have yielded negative results [135]. 

### 4.2. Vesicle-Mediated Transport/Endocytosis

A second-pathway was endocytosis. This is supported by five genes (*PICALM; SORL1; APOE; BIN1; LDLR*), and it appears to be involved in APP trafficking. Alterations in the intracellular transport of APP can directly influence whether APP undergoes *α*-secretase enzymatic activity, releasing a nontoxic peptide, *α*-secretase-cleaved soluble APP (sAPP*α*), or follows *β*-secretase and *γ*-secretase enzymatic pathways, leading to generation of the neurotoxic forms of beta amyloid. While the *α*-secretase enzymes are found at the cell surface, *β*-secretase lies within the Golgi apparatus and endosomes, the *γ*-secretase complex in the endoplasmic reticulum, lysosomes, and the cell surface. When APP is moved into the endosome, it is cleaved by *β*-secretase and then transported either to the cell surface or to the lysosome to be further processed by *γ*-secretase to form beta amyloid. However, when APP accumulates at the cell surface, it has a greater chance of interacting with *α*-secretase to form nonamyloid-forming sAPP*α* [136, 137]. 

### 4.3. Immune System

Seven genes (*TNF; IL8; CR1; CLU; CCR2; PICALM; CHRNB2*) were found to interfere with the immune system. Neuroinflammation is considered to be a downstream consequence of amyloidogenesis. Beta-amyloid deposition within the CNS would bring about the activation of microglia and thus initiate a proinflammatory cascade leading to release potentially neurotoxic substances (cytokines; chemokines; reactive oxygen and nitrogen species; proteolytic enzymes) and to amplify neural damage [138]. It has also been suggested that activated microglia may lead to phosphorylation of tau and formation of neurofibrillary tangles [139, 140]. Based on inflammatory damage, a number of randomized trials compared the efficacy of nonsteroidal anti-inflammatory drugs and COX inhibitors as antidementia treatments but they yielded negative results [141]. Inflammatory cells can also mediate the clearance of beta amyloid via phagocytosis [142, 143]. This has suggested that increased proinflammatory cytokines and activated microglia in AD patients may be compensatory for defective clearance of beta amyloid on one hand, and this inflammatory cascade may cause brain damage on the other hand [144]. Other pathways emerging from GO analysis were less frequently discussed. Five genes were implicated in alcohol metabolism. The relationship between alcohol consumption and dementia is dose dependent. Alcohol abuse was associated with increased prevalence of cognitive dysfunction in the elderly, whereas a daily alcohol consumption of less than 40 g for women and 80 g for men was protective against cognitive impairment [145]. The protective effect of moderate drinking was confirmed in prospective studies [146]. Instead, heavy drinking usually leads to cognitive disorders, but brain lesions [147] as well as cognitive deficits [148–150] are different in alcohol-related dementia and AD. *APOE* was found to modulate the link between alcohol and AD [151]. In particular, the impact of alcohol on brain appears to be more detrimental in APOE epsilon4 carriers [152, 153].

### 4.4. Genetic Networks

These pathways are actually interconnected. One such network is lipoprotein-inflammation apoptosis. Central links in this chain are *APOE* and *CLU*. Animal models have shown the influence of APOE alleles on proinflammatory cytokine (TNF-alpha; IL-6; IL-1) expression and sepsis [154–156]. Recently, we have reported an association between APOE alleles and IL-1beta levels in patients with AD [157]. Apolipoprotein E has a protective effect against apoptosis which is significantly reduced in the presence of the pathogenic epsilon 4 isoform [158]. Clusterin that is involved in cholesterol metabolism (it is also named apolipoprotein J) and the regulation of complement cascade is known to block apoptosis by binding to proapoptotic mediator Bax and sequestering it in the cytoplasm, thereby, preventing Bax-triggered mitochondrial apoptosis [159]. A second network is centered around intracellular transport of APP, and macroautophagy intracellular transport is mediated by endocytic pathway. Sorting of internalized molecules occurs in the early endosome, which directs the material back to the plasma membrane for recycling, to the trans-Golgi network for further processing, or to late endosomes and lysosomes for degradation. APP potentially undergoes processing at each of these locations. Early endosomes produce A**β** from APP in normal cells and mediate the uptake of A**β** and soluble APP [160]. Beta-amyloid localization to enlarged endosomes is prominent in early developmental AD [161, 162]. Macroautophagy is a constitutively active branch of the wider endosome-autophagosome-lysosome system, involved in the sequestration of cytosolic regions into characteristic double-membrane or multimembrane autophagosomes that are delivered to lysosomes for degradation [163]. Macroautophagy interferes with different stages of APP-beta-amyloid cycle, and it affects both APP proteolysis to beta amyloid [164] and lysosomal proteolysis in postsecretase APP catabolism [165]. Autophagic vacuole are identifiable by the protein, LC3-II (phospho-lipidated form of microtubule-associated protein 1 light chain 3-I, MAP1 LC3-I), which is associated with both luminal and cytosolic surfaces of vacuole membranes [163]. Classical autophagy activation is regulated through PI3 K/Akt/mTOR pathways although alternative mTOR-independent pathways also exist [166]. The endocytic and autophagy pathways converge onto the lysosom system. Beta amyloid is generated in multivesicular bodies of the endosomal pathway and may also be generated in autophagosomes [167]. In recent years, several genes could be linked to LOAD from large genome-wide association studies (GWASs) [168]. However their effect sizes are small (OR 1.20–1.66), consistent with those reported for other neuropsychiatric disorders [169], and most genetic variability is still unexplained. Using a method similar to the present one, authors clustered functionally interrelated genes, and they tested such networks in ninety-six heritable disorders. This allowed to detect at least one disease gene in 54% of the loci studied, representing a 2.8% increase over random selection of candidate genes [170]. This suggests that reconstructing shared functional pathways may significantly reduce the cost and effort of pinpointing true disease genes in disorders for which multiple susceptibility loci have been reported. On the other hand, analyzing complex networks of genes that are altered in AD patients by means of genomics or proteomics, it is possible to dissect them into clusters, each associated with a specific biological pathway. Such clusters could then be investigated in single patients with AD who are pathophysiologically heterogeneous although they share the same diagnostic label. This would provide more suitable targets for AD treatment. 

### 4.5. Secondary Genetic Effects

Several characteristics of AD patients, not merely diagnostic identification, are affected by AD genes [86]. These secondary effects should be incorporated to refine genetic networks. A well-known epidemiological finding is that AD is more prevalent in women [171, 172] although authors have contended that this could be an age effect [1]. A connection may exist between gender and APP trafficking. In fact, a few studies have shown that the association of sorting protein genes *SORL1* and *SORCS1* with LOAD was limited to women [67, 75, 123]. Similarly, a few haplotypes of LDLR polymorphisms were more represented in AD patients and associated with altered biomarkers (CSF Abeta(42); tau protein) in women [98]. Notably, estrogen increases APP transport within the trans-Golgi network [173]. A large subset of patients with AD (60%–80%) have neuropsychiatric symptoms such as depression, agitation, and psychosis (behavioral and psychiatric symptoms of dementia, BPSD) [174, 175]. These disturbances are associated with worse prognosis, more rapid cognitive decline, higher costs of care, increased caregiver burden and earlier nursing home placement. Proinflammatory cytokines may play a significant role in BPSD. In fact, the C-511T polymorphism in the promoter region of the IL-1 beta gene was found to correlate with depressive and psychotic symptoms in AD patients [176, 177]. Similarly, a IL-1alpha SNP (rs1800587) was associated with AD-related depression [178]. Genetic variations at *SORL1* may be associated with AD-related psychosis as well although this is still controversial. In fact, one published study revealed no association between *SORL1* polymorphisms and AD psychosis [179]. On the contrary, we found that SNPs 8–10 were associated with psychotic manifestations in AD patients [75]. Among possible endophenotypes, *SORL1* was associated with a selective deficit in abstract reasoning [180] and MRI changes [60]. Other genes have been less extensively investigated. *CST3* was found to correlate with age of onset in sporadic AD [181] as well as EEG alterations in subjects with AD and mild cognitive impairment [182]. Plasma clusterin concentrations were associated with brain atrophy, severity of Alzheimer's disease, and rate of clinical progression although there was no effect of *CLU *SNPs on gene and protein expression [36].

### 4.6. Gene-Gene Interactions and Epigenetics

Gene-gene interactions may account for a substantial genetic variability in LOAD. Gene-gene interactions were reported between *IL6* and *IL10 *[183],* IL6* and *A2M *[184], and *IL1A *and *IL8*.* APOE *was found to interact with genes encoding methylenetetrahydrofolate reductase (*MTHF*) [185], luteinizing hormone receptor [186], and angiotensin-converting enzyme (*ACE) *[187]. To increase the number of gene-gene interactions, a useful approach could be to investigate genetic networks that are based on homogeneous biological pathways. Epigenetic modifications alter the structure of chromatin to influence gene expression. A common epigenetic pathway is DNA methylation. This occurs naturally on cytosine bases at CpG sequences, and it is usually associated with triggering histone deacetylation, chromatin condensation, and gene silencing. Differentially methylated cytosines give rise to distinct patterns specific for each tissue type and disease state. Among AD genes, *PSEN1*, *APP, *and *APOE* have abundant CpG sites and are significantly affected by methylation [188]. Decrements in markers of DNA methylation were consistently reported in AD neurons and could explain discordant AD onset in twin pairs [189]. Developmental exposure to xenobiotics such as lead (Pb) influences methylation in AD genes, and this would predispose to AD later in life [190]. 

### 4.7. Pharmacogenomics

Response to antidementia drugs is also affected by genetic factors. Pharmacogenomics in AD is still in its infancy, with genes associated with AD pathogenesis and genes responsible for drug metabolism (cytochrome P450) [191]. In monogenic-related studies, APOE-4/4 carriers are the worst responders. In trigenic (APOE-PS1-PS2 clusters)-related studies, the best responders are those patients carrying the 331222-, 341122-, 341222-, and 441112-genomic profiles. The worst responders in all genomic clusters are patients with the 441122+ genotype. This would indicate a powerful effect of APOE genotypes on therapeutics in networking activity with other AD-related genes converging on the same biological pathways. 

## 5. Conclusions

This review was based on the most comprehensive collection of published studies about the genetics of AD. The best genes were classified according to qualitative criteria such as size of research samples, heterogeneity across studies, and control for various sources of bias including small effect size (OR) and publication bias. However, there were also important limitations, mainly due to Alzgene design. First, meta-analyses were restricted to allele contrast, which is less powerful than genotype-based test and allows no inference of the true underlying mode of inheritance, and there was no genetic information at haplotype level. Moreover, only the main effect was investigated, that is diagnostic association with AD, while other clinical phenotypes and endophenotypes could not be considered alongside gene-gene and gene-environment interactions. On the contrary, a nonnegligible effect of LOAD genes may be directed to these secondary targets as suggested elsewhere. Gene ontologies were developed to provide a shared representation of genes and gene products across species. GO terms contain broad definitions of biological processes in the living cell. Hence, these terms are suitable to identify areas for genomic exploration (e.g., all genes implicated in cholesterol metabolism) but not to elucidate pathogenic mechanisms in depth. 

Notwithstanding these caveats including all published studies in a single open-access database (Alzgene) highlights the most important pathophysiological mechanisms, which show the convergence of many genes, and it more easily prompts new biological hypotheses. 

## Figures and Tables

**Table 1:** Top-rated genes associated with LOAD.

Gene	Ch	N° minor	Quality (HuGENet)	Caucasian OR	Asian OR	All ethnic groups OR
APOE	19	4,167	AAA	3.77 (3.29–4.32)	3.99 (2.86–5.57)	3.61 (3.20–4.08)
CLU	8	53,712	AAA	0.87 (0.85–0.90)	n.a	0.88 (0.86–0.91)
PICALM	11	44,358	AAA	0.89 (0.86–0.92)	n.a	0.90 (0.86–0.93)
EXOC3L2	19	13,519	AAA	1.17 (1.12–1.23)	n.a	1.17 (1.12–1.23)
BIN1	2	24,713	AAA	1.14 (1.08–1.21)	n.a	1.14 (1.08–1.21)
CR1	1	18,779	AAA	1.14 (1.08–1.20)	n.a	1.16 (1.09–1.22)
SORL1	11	1,734	AAA	1.07 (1.00–1.15)	1.30 (1.13–1.50)	1.10 (1.02–1.17)
TNK1	17	3,538	AAA	0.84 (0.76–0.93)	n.a	0.84 (0.76–0.93)
IL8	4	1,157	AAA	1.26 (1.01–1.58)	n.a	1.26 (1.01–1.58)
LDLR	19	1,228	AAA	0.85 (0.72–0.89)	n.a	0.85 (0.72–0.89)
CST3	20	1,203	AAA	1.28 (1.04–1.56)	n.a	1.23 (1.03–1.48)
CHRNB2	1	227	BAA	0.69 (0.51–0.95)	n.a	0.67 (0.50–0.90)
SORCS1	10	567	BAA	1.34 (1.09–1.65)	n.a	1.34 (1.09–1.65)
TNF	6	301	BAA	n.a	1.37 (1.05–1.79)	1.35 (1.39–1.77)
CCR2	3	308	BAA	0.73 (0.56–0.97)	n.a	0.73 (0.56–0.97)

OR values are referred to the best SNP for each gene.

n.a: one study or none; meta-analysis could not be performed.

HuGENet classification was used to assess the quality of studies (see text).
